# Atypical Mycosis in Psittacine Birds: A Retrospective Study

**DOI:** 10.3389/fvets.2022.883276

**Published:** 2022-05-12

**Authors:** Livio Galosi, Christian Falcaro, Patrizia Danesi, Claudia Zanardello, Sara Berardi, Lucia Biagini, Anna-Rita Attili, Giacomo Rossi

**Affiliations:** ^1^School of Biosciences and Veterinary Medicine, University of Camerino, Matelica, Italy; ^2^Laboratory of Parasitology, Istituto Zooprofilattico Sperimentale delle Venezie, Legnaro, Italy

**Keywords:** psittacine birds, atypical mycosis, *Mucor racemosus*, *Mucor circinelloides*, *Alternaria* spp., *Fusicladium* spp.

## Abstract

A retrospective study was conducted on parrots submitted from necropsy to the Department of Veterinary Pathology, School of Biosciences and Veterinary, University of Camerino, Italy, from 2007 to 2018. From a total of 2,153 parrots examined at post-mortem, four cases were diagnosed with atypical mycosis and were considered for determination of the fungus species by PCR. A Fischer's lovebird (*Agapornis fischeri*), Peach-faced lovebirds (*Agapornis roseicollis*), and two Blue and Gold Macaws (*Ara ararauna*) from four different aviaries died after some days of lethargy and ruffled feathers. Records of gross necropsy and histopathological exams (H&E, PAS, and Grocott stain) were described and biomolecular analyses were carried out. No specific gross lesions were appreciated at necropsy, while histopathology evidenced a systemic mycosis in several organs, particularly in the lungs. In affected organs, broad and non-septate hyphae, suggestive of mycoses, were observed. Molecularly, *Mucor racemosus* (Fischer's lovebird) and *M. circinelloides* (Peach-faced lovebirds) were identified from formalin-fixed and paraffin-embedded (FFPE) lung and liver tissue. In addition, *Alternaria alternata* and *Fusicladium* spp. (respectively in male and female Blue and Gold macaws) were identified in FFPE tissue from several organs; whereas the role of *Mucor* spp. as true pathogens is well-demonstrated, and the behavior of *A. alternata* and *Fusicladium* spp. in macaws as opportunistic pathogens have been discussed. To our knowledge, this report is the first one reporting mucormycosis caused by *M. racemosus* and *M. circinelloides* in lovebirds, and *A. alternata* and *Fusicladium* spp. in macaws.

## Introduction

In healthy individuals, fungi rarely cause disease; nevertheless, opportunistic and pathogenic fungi can cause severe disease when immunosuppression or other debilitating conditions affect the host (1). Aspergillosis is the most common fungal disease in birds, mostly caused by *Aspergillus fumigatus* and *A. flavus*, although the genus includes more than 300 known species (1–4). In birds, *Aspergillus* spp. tends to initially colonize the lower respiratory tract and the infection can develop as a focal aspergilloma or disseminate to different organs (5). Another very common fungal disease in birds is candidiasis, which is caused by fungi of the genus *Candida* that are frequently found in the microbiota of healthy humans and animals and, thus, considered commensal and facultative pathogens. Infections in the oral cavity and gastrointestinal system are the most common forms of candidiasis in birds (1, 6).

*Cryptococcus* species can also cause invasive diseases of the upper respiratory system of parrots kept in captivity (7–11). This can result in clinical signs referable to mycotic rhinitis or involve anatomic areas closely connected with the nasal cavity, like the retrobulbar space, palate, choana, sinuses, and beak (7, 8). In some instances, the infection involves bones causing osteomyelitis (9, 10).

More rarely than the previous orders of fungi, Mucorales represents a group of fungi living as saprotrophs in soil, decaying organic and dead plant material, and able to cause disease in a wide variety of vertebrates (12). Although the order Mucorales was considered problematic for the nomenclatural stability due to a satisfactory identification using mostly the microscopic morphology, a clear phylogenetic classification was recently proposed using DNA barcoding, introducing taxonomic nomenclatural changes in this group (13).

Likewise, to candidiasis and aspergillosis, a deficiency of the immune system or metabolic disorders can act as predisposing factors to Mucorales infection (1). Few reports are published on the mucormycosis in avian species, in which the respiratory and gastrointestinal tract are mainly involved (14–16). Alongside that most important fungal genus, additional groups of fungi can cause disease in humans and animals, such as the opportunistic mold *Alternaria* spp. The latter represents a parasite of plants (17), but it is also often involved in dermatomycosis in animals (18), resulting in cutaneous nodular lesions in horses (19). In addition, the genus *Fusicladium*, usually not involved in vertebrate pathology, could be an unintentional discovery in animal clinical cases. The present article describes four pathological cases in which opportunistic fungi are identified as the cause of death in psittacine birds.

## Materials and Methods

### Animals

A retrospective study was conducted on parrots submitted for necropsy to the Department of Pathology, School of Biosciences and Veterinary, University of Camerino, Italy, from 2007 to 2018. From a total of 2,153 necropsied parrots that were present in the archive, four cases received the histological diagnosis of atypical mycosis. These cases, involving a 10-year-old male and a 17-year-old female of Blue and Gold Macaw (*Ara ararauna*), a 1-year-old male Fischer's lovebird (*Agapornis fischeri*), and a 2-year-old female Peach-faced lovebird (*Agapornis roseicollis*), coming from different Italian aviaries, were considered for determination of the fungus species by PCR. For all birds, the anamnesis reported some days of lethargy and ruffled feathers before death, without particular clinical signs.

### Pathology

After necropsy, tissue samples were fixed in 10% neutral buffered formalin, routinely processed, and embedded in paraffin wax. Three-μm thick histological sections were stained with hematoxylin and eosin (H&E), Periodic Acid Schiff (PAS), and Grocott histochemical stains.

### Molecular Investigations

To genetically characterized fungal elements, at least five formalin-fixed and paraffin-embedded (FFPE) tissue sections (5–8 μm) of lung and liver (for the Fischer's and Peach-faced lovebirds) or pool of organs (for the Blue and Gold Macaws) were sent to the Parasitology Laboratory of the “Istituto Zooprofilattico Sperimentale delle Venezie,” Italy, for molecular investigations.

A single slice was transferred to a 1.5-ml tube and DNA extraction was performed by using ReliaPrep^TM^ FFPE gDNA Miniprep System (Promega), including negative control (20). Each extraction was performed in duplicate.

The DNA was amplified by using SYBR Green Real-Time PCR (rtPCR) with three sets of primers targeting a short portion of the ITS 1 region (*i*), and 2 portions of the LSU rRNA (*ii* and *iii*).

i. Primers ITS5 (21) and the in-house designed primer ITS288 (5′-AAG AGA TCC GTT I GAA AG-3′). (Amplicon length 190–210 bp).
ii. Primers NL1/NL4 (22) targeting a portion D1/D2 domain of the 28S rRNA (Amplicon length 600–650 bp).
iii. Primers 12F/13R (23) targeting the extended region of the 28S rRNA (Amplicon length 200–230 bp).

The rtPCR reaction was conducted according to a standardized protocol (20, 24). All amplicons were sequenced for fungal identification by using Blast in the GenBank database. Sequencing reactions were performed from both ends. Alignment was performed with ClustalW integrated into MEGA v6.0 (25), and then, was manually refined. Phylogeny was performed using the Neighbor-joining method on the 28S LSU ribosomal RNA (rRNA) sequence dataset. We added sequences of Zygomycetes including *Mucor, Rhizopus, Saksenaea, Apophysomyces*, and *Lichtheimia* species available from GenBank. The sequence of the *Syncephalastrum monosporum* was used as an outgroup.

## Results

### Necropsy and Histopathological Examination

Poor body conditions were recorded in all the birds but, at the opening of the coelomatic cavity, no specific gross lesions were appreciated. In all parrots, various areas of acute suppurative inflammation with focal granulomatous lesions were observed in the gastrointestinal tract and the liver. Lungs were characterized by acute congestion and some areas of parenchyma consolidation. Histopathology evidenced a systemic mycosis with different degrees of fungal vasculitis, angioinvasion, and, in some cases, intravascular thrombosis was observed. A focally marked inflammatory response, represented by heterophils, enriched exudate, and scattered granulomatous lesions, was observed in lovebirds. In Blue and Gold Macaws, the minimal inflammatory response described suggested that the birds were most likely immunosuppressed.

### Fischer's Lovebird (*Agapornis fischeri*)

**Figure 1:**
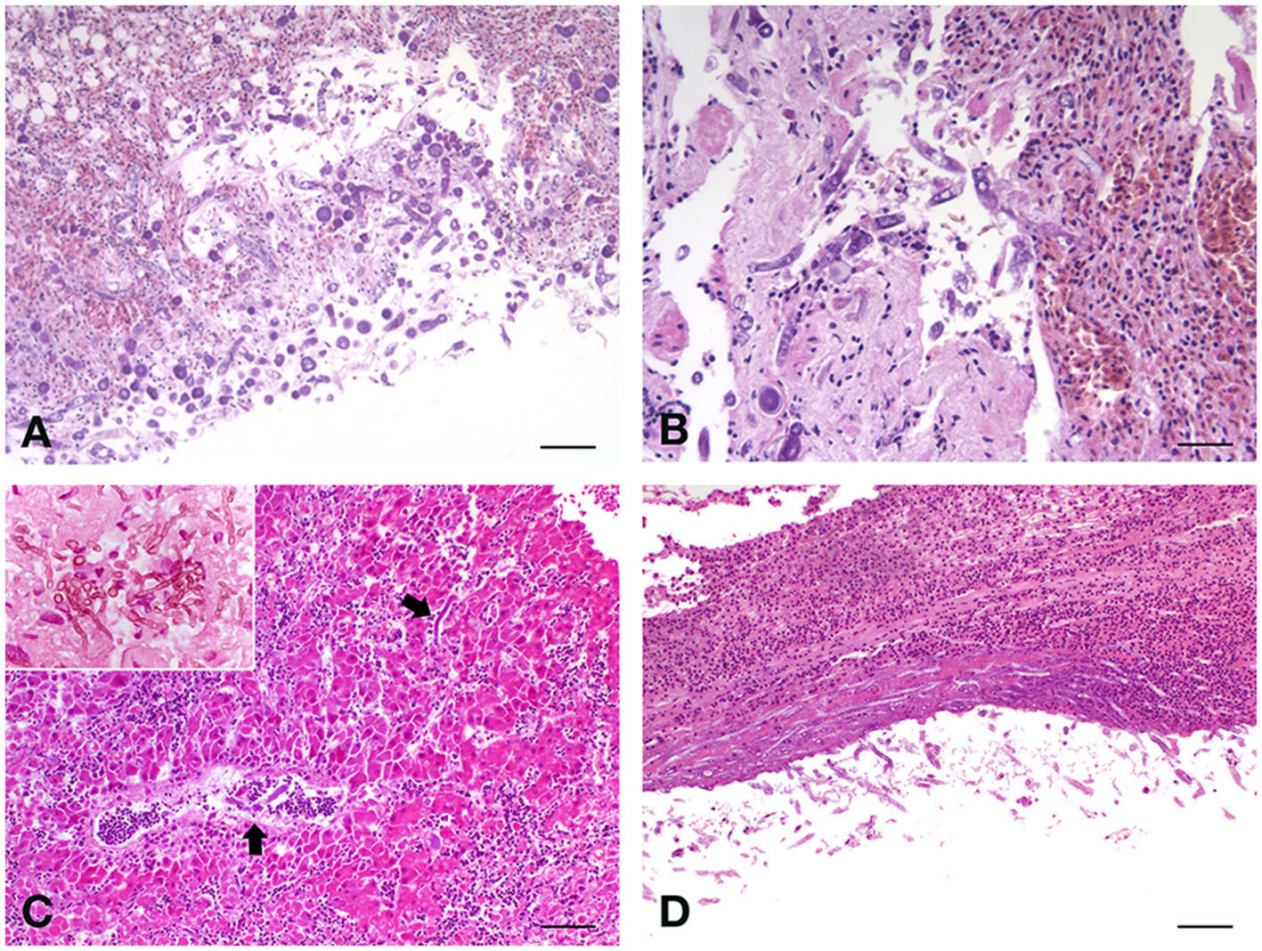
**(A)** Fischer's lovebird (*Agapornis fischeri*). Lung parenchyma with the invasive presence of poorly septate large mycotic hyphae, with large sporangiophore, simply spherical and hat-shaped, in the absence of rhizoids relative to the sporangiophores. H&E, scale bar = 250 μm. **(B)** Peach-faced lovebird (*Agapornis roseicollis*). Lungs characterized by acute congestion and some areas of parenchyma consolidation, with the presence of mycotic hyphae. H&E, scale bar = 100 μm. **(C)** Blue and Gold macaw (*Ara ararauna*), male. Branching septate fungal hyphae were observed in the liver. H&E, scale bar = 250 μm. Insert, scale bar = 50 μm. **(D)** Blue and Gold macaw (*Ara ararauna*), female. Long, septate, branched hyphae were observed also in the kidney, both at the level of the serosa and in the parenchyma. H&E, scale bar = 250 μm.

Histopathological analysis revealed chronic-active pyogranulomatous hepatitis, associated with very abundant coagulative necrosis of large parenchymal areas. Granulomas, with a necrotic center surrounded by a sporadic fibrotic reaction and a wall consisting of epithelioid cells, are adjacent to the necrotic areas, contain a large uniseptate or pauci-septate hyphae, irregular in shape, and suggested an infection sustained by Mucorales, as these hyphae differ from those of *Aspergillus* spp., that are septate, dichotomous, and branching hyphae. The hyphal elements are usually found surrounded by abundant necrosis, hemorrhagic areas, and thrombosis of blood vessels (26). In some necrotic areas, the fungi invade the blood vessel wall or are found inside the lumen and extensive lymphoplasmacellular reactions circumscribe the hepatic nodules. The splenic parenchyma is characterized by notable perivasculitic phenomena and extensive necrotic process, with a high percentage of apoptotic lymphocytes and with evident microgranulomas, one of which is very well-structured and with the center with extensive necrosis. Also here, there are fragments of septate hyphae in the central area of a larger granuloma. Lungs appear affected by severe chronic pneumonia with strong collectivization of the parabronchial septa and parenchymal carnification, with large overlying serositis. The mycotic colonization with circumscribing inflammatory phenomena is severe (Figure 1A). Also, in the lungs, as well as in the liver, discrete and poorly encapsulated granulomas are observed, among diffuse fibrovascular tissue. T cells are the predominant infiltrating lymphoid cells in these lesions, suggesting the importance of cell-mediated response to infection. Air sacs are characterized by a widespread and evident form of inflammation and degeneration of mesothelial cells.

### Peach-Faced Lovebird (*Agapornis roseicollis*)

Severe alterations were observed also in the liver of this bird, characterized by areas of cariorectic necrosis, with diffuse fragmentation of the involved cells. Large, irregular, and pauciseptate fungal hyphae were observed, usually immersed in abundant necrotic material, and surrounded by hemorrhagic areas, in which a heterophilic exudate was constantly present. Also, in this case, blood vessel thrombosis, involving frequently the central-lobular vein, was observed, with fungal elements invading the vessel wall and heterophils and info-plasma cellular enriched infiltrate surrounding the lesions. In general, diffuse hepatocellular degeneration was observed in the remaining areas that are not directly involved in the necrotic-inflammatory processes. In the kidney, diffuse urate precipitates were evident within the tubular structures, and massive infiltration of the same giant and poorly septate hyphae, both at the level of the serosa and the parenchyma, were observed. Lung parenchyma appeared involved by strong diffusive pyogranulomatous exudate, with fibrovascular tissue formation at the interstitial level. The invasive presence of poorly septate large mycotic hyphae was enriched by some large spherical sporangiophore, without rhizoids (Figure 1B). The epithelium of parabronchi was necrotic, and the lumen was replete with eosinophilic exudate. Few spherical sporangiophores were present in pulmonary lesions.

### Blue and Gold Macaw (*Ara ararauna*), Male

The proventriculus of this parrot was characterized by a severe necrotic and granulomatous process, with severe mucosal erosion and ulceration, with extensive colonization of numerous spores and septate hyphae in the mucosal area, evidencing the presence of round to oval, and with thick-walled fungal structures representing large muriform conidia with tapering apices, with numerous fragments of septate hyphae. The liver showed a diffuse hepatocellular degeneration with a deeply extending granulomatous pattern, also showing areas of necrosis. In these granulomas, pseudo-epitheliomatous hyperplasia was also seen, with an inflammatory infiltrate constituted by histiocytes with multinucleated giant cells, and numerous heterophils. At high magnification, branching septate fungal hyphae were also observed, characterizing this form of mycosis (Figure 1C). Interestingly, the spleen of this parrot showed a very strong depletion of the white pulp, which is almost absent, indicating a profound immunosuppressive state of the bird. No lesions were observed in the lungs, air-sacs, and kidneys.

### Blue and Gold Macaw (*Ara ararauna*), Female

As in lovebirds, the most affected organ in this macaw was the liver, showing severe morphological damage consisting of a diffuse hepatocellular degeneration, and the hepatocyte chains were infiltrated by chronic inflammatory cells (lymphocytes, plasma cells, and giant cells). Scattered, thick-walled, and multiseptate muriform cells, measuring 6–12 μm and divided by fission were evidenced, bringing to the diagnosis of chromoblastomycosis. Long, septate, branched, and strongly PAS-positive hyphae were also observed in the kidney (Figure 1D), both at the level of the serosa and in the parenchyma, with diffuse urate precipitates within the tubular structures. Lungs and air sacs showed the massive presence of septate hyphae, smooth thin-walled, guttulate, branched, 2–3 μm wide, and with conidiophores either micronematous or semi-macronematous. The colonized pulmonary parenchyma was involved in a strong pyogranulomatous and poorly encapsulated inflammatory reaction, enriched by heterophils, eosinophils and abundant macrophages, plasma cells, and lymphocytes. Starting from interstitial areas, fibrovascular tissue was diffusely produced inducing atelectasis areas.

### Molecular Investigations

Positive DNA amplification was obtained from FFPE tissue from all 4 birds, with ITS (*i*) and 28S rRNA (*ii*) rtPCR protocols. The 12F/13R (*iii*) rtPCR amplified DNA only from *Agapornis* tissues.

For the lovebirds, all amplicons were successfully sequenced and *Mucor racemosus* (MT240480) and *M. circinelloides* (MT240488) were identified in *Agapornis fischeri* and *Agapornis roseicollis*, respectively with a similarity of 100% when blasted in GenBank database.

In the two Blue and Gold Macaws, only a portion of the 28S rRNA amplicons (*ii*- NL1/NL4 rtPCR) was successfully sequenced. *Alternaria alternata* (in the male) and *Fusicladium* spp. (in the female) were identified with 98% and 100% similarity, respectively. The sequencing of ITS amplicons was not possible, or sequences were of poor quality, showing double peaks in the electropherograms.

**Figure 2:**
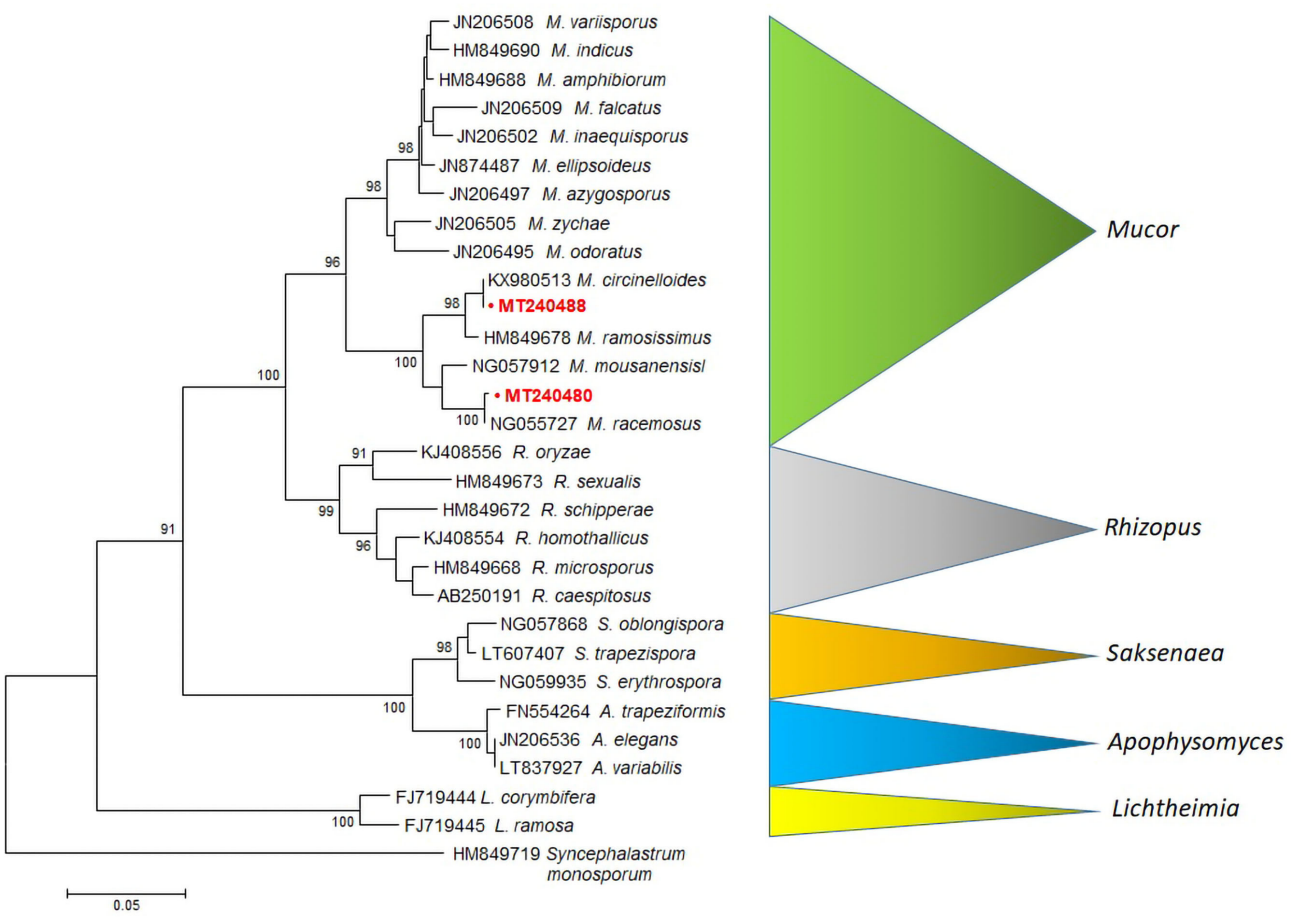
MEGA6 neighbor-joining tree of the Mucorales and related species (*Mucor, Rhizopus, Saksenaea, Apophysomyces* and *Lichtheimia*) based on LSU sequences (28S rRNA). Bootstrap values shown at the main nodes represent the probabilities based on 1,000 replicates. Sequence of *Syncephalastrum monosporum* was used as an outgroup. The sequences produced by our study are highlighted in bold red.

A rooted tree was constructed with 28S LSU rRNA *Mucor* sequences obtained from *Agapornis* birds. Sequences of *M. racemosus* (MT240480) and *M. circinelloides* (MT240488), clustered into highly supported (bootstrap value = 100%) clades, are clearly separated from *Mucor, Rhizopus, Saksenaea, Apophysomyces*, and *Lichtheimia* species (Figure 2).

## Discussion and Conclusion

This study describes unusual fungal findings in four parrots. The molecular characterization confirmed the histological description and identified two *Mucor* species (*M. racemosus* and *M. circinelloides*) as most likely true pathogens in the two lovebirds. The evidence of *Alternaria* and *Fusicladium* in Blue and Gold Macaw, suggests that saprophytic fungi can act as opportunistic pathogens in some instances, and are mostly linked to an immunodeficiency of the host.

Fungi are ubiquitous organisms both as saprophytes in the environment or commensal in the host (27). “Commensalism” is the state of infection that causes no damage or clinically unapparent damage to the host, though it can arouse a response of the immune system (28). Although evolution has provided both the parasites and the host's immune system with similar mechanisms of selection and defense (29), sometimes the interplay with some microorganisms is deleterious for the host and, consequently, opportunistic infections can take place (30). Several fungal genera, including *Alternaria* spp., *Aspergillus* spp., *Mucor* spp., and *Venturia* spp. were isolated from migratory birds, confirming the role of birds as a possible vector of fungi and the commensalism of fungi with their avian host (31–33).

Most species of the numerous fungi groups are saprobic in several environments (34). *M. racemosus* and *M. circinelloides* are ubiquitous fungi belonging to the phylum Zygomycota and order Mucorales. Like most fungi, they can be found in healthy birds (35), but are considered the primary pathogen of severe diseases, known as zygomycosis or mucormycosis, in case of immunodeficiency of the host (36). *M. circinelloides* and *M. racemosus*, which were identified as the cause of death in two lovebirds in this study, were isolated in different types of soils in Mexico, France, and Iran (34). Interestingly, excreta of herbivore animals, including most of the psittacine birds, are rich in digested plant material and can host a wide variety of fungal strains (34). For this reason, for example, poultry-house litter shows a severe increase in fungal presence after the completion of the birds' growth cycle (37). It is, therefore, reasonable to hypothesize that poor hygiene in the cage of captive-bred birds can cause strong contamination of fungal elements in their living environment and increase the possibility of infection. Lovebirds are traditionally kept in small cages and usually, the breeding pair fills a wooden nest with plant material, spending all the night and most of the day inside this nest (38). All the aforementioned elements promote the overgrowth of fungi. Furthermore, predisposing factors, as well as the debilitation of each individual, are fundamental for the development of infection by fungal flora. Thus, efforts should be directed to avoid stress in the aviaries or cages and strict hygiene should be encouraged (27).

In humans and animals, there are three basic exposure pathways to pathogenic fungal infection: inhalation, ingestion, and direct contact, in case of disruption of the cutaneous barrier (34). In human medicine, mucormycosis became the third most common invasive mycosis in order of importance, after candidiasis and aspergillosis (36). In birds, the most common mycosis is aspergillosis, caused by *Aspergillus fumigatus* in 95% of cases. Infection is favored by the host species, with a predilection for turkeys, penguins, raptors, and waterfowl, as well as by environmental conditions, immunosuppression, physical stress (migration), and administration of exogenous corticosteroids (5). Fungi of the genus *Candida* spp. are the second most common agents of mycosis in birds, typically affecting the gastrointestinal tract (6, 39). Other fungi are rarely described as pathogens in birds.

The *M. racemosus* has been identified as a pathogen in immunocompromised human patients (40), as well as in birds (16), where it has been associated with circovirus infection, which normally induces immunosuppression. However, in the 4 cases described in this article, no inclusion bodies or other lesions referable to viruses were found, and the isolated fungi were identified as a primary pathogen, actually causing the death of the birds. Mucorales species have a vascular tropism, causing tissue infarctions, and they can lead to cutaneous, rhinocerebral, and sinopulmonary diseases, which in turn can evolve in disseminated and fatal infections, especially in immunocompromised hosts (36). In our cases, the fungi most likely entered through the respiratory tract, given the constant involvement of the lungs and air sacs found at the necroscopic examinations. Then, the pathogens generalized through the bloodstream, causing vascular lesions and widespread areas of inflammation at the level of highly vascularized organs with filter function, such as the liver and kidneys. Only in the case of the Blue and Gold Macaw male, in which the gastrointestinal tract was diffusely affected with large ulcerative lesions and the involvement of the vessels of the submucosa, it is possible to hypothesize a different mechanism of entry of the fungi, with a primary localization to the liver through the portal route. The predilection of fungi for the respiratory entry route in birds and some classes of reptiles can certainly benefit from the particular anatomy of the respiratory system of these animals due to the presence of air sacs. This, and some other anatomical features, preclude the mechanisms of ejection of inhaled fungal spores that remain in areas of low turbulence of the airflow, such as parabronchi and air sacs, where constant temperature and humidity favor spores' germination. In addition, the absence of resident macrophages within airway lumens and the dependence on heterophils (that use cationic proteins, hydrolase, and lysozyme rather than catalase and myeloperoxidase) could also be liable for the increased susceptibility of birds to develop lesions of mycotic origin that can be devastating (41, 42).

*Alternaria* spp. are plant parasites, causing leaf spots on adult plants and wilt diseases of sprouting seeds. Some species, such as *A. alternata* and *A. tenuissima*, are saprophytic on a wide range of decaying plant tissues (17) and consequently are very common on every animal farm, where they can contaminate the animals' skin with spores (19). In horses, *A. alternate* causes cutaneous nodular dermatitis (19, 43, 44). *Alternaria* spp. can also infect cats (45–48) and less commonly dogs. A case report in the latter species describes *A. infectoria* as the cause of multiple cutaneous lesions in a patient under immunosuppressive therapy for immune-mediated hemolytic anemia (49). In birds, infection of *Alternaria* spp. is rarely reported. In Indian jungle bush quail (*Perdicula asiatica*), *A. alternata* causes a season-dependent lung invasion, causing severe disease when the lowest immune status occurs (50). In psittacine birds, only a case is reported, describing a dermal infection of *A. alternata* in an Indian ring-necked parakeet (*Psittacula krameri*), with feather picking and a skin lesion on the wing (51). *A. alternata* was isolated in decaying nests of wild birds, made up of twigs, leaves, and bark, which are all substrates decomposed by fungi (52, 53). This observation reinforces the hypothesis that poor hygiene in the management of the captive birds included in this study could have had an important role in the pathogenesis of mycosis.

*Fusicladium* spp. (anamorph of *Venturia*) are phytopathogens causing worldwide significant economic loss to crops (54). According to molecular analysis, ascomycetes, such as *Dothideales* / *Capnodiales* (for example, *Pseudocladosporium, Fusicladium*), are anamorphs and morphologically similar to *Cladophialophora*, were reclassified, with the dothidealean species *Venturia hanliniana* classified as the teleomorph of *Fusicladium brevicatenatum* (55). Another distinction between *Chaetothyriales* and *Dothideales*/*Capnodiales* is found in their ecology, with opportunistic infections and lesions in humans. Some of these organisms, attributed to *Cladophialophora* and predominantly saprobic or plant-associated, were never associated with infection in vertebrates.

To our knowledge, this report is the first one describing *M. racemosus* and *M. circinelloides* infections in lovebirds, and *A. alternata* and *Fusicladium* spp. in macaws. Although infections caused by atypical fungi in parrots are rare, exposure to these pathogens should always be considered by clinicians during clinical practice and aviary management.

## Data Availability Statement

The datasets presented in this study can be found in online repositories. The names of the repository/repositories and accession number(s) can be found in the article/supplementary material.

## Author Contributions

LG, CF, PD, and GR conceived the study. LG, CZ, LB, and GR performed necropsies and histological analysis and wrote the manuscript. CF and PD performed molecular analysis and wrote the manuscript. SB, A-RA, and GR reviewed the article and provided critical suggestions and comments. All authors discussed the results and approved the final manuscript.

## Conflict of Interest

The authors declare that the research was conducted in the absence of any commercial or financial relationships that could be construed as a potential conflict of interest.

## Publisher's Note

All claims expressed in this article are solely those of the authors and do not necessarily represent those of their affiliated organizations, or those of the publisher, the editors and the reviewers. Any product that may be evaluated in this article, or claim that may be made by its manufacturer, is not guaranteed or endorsed by the publisher.
